# Coronarin K and L: Two Novel Labdane Diterpenes From *Roscoea purpurea*: An Ayurvedic Crude Drug

**DOI:** 10.3389/fchem.2021.642073

**Published:** 2021-04-21

**Authors:** Venugopal Singamaneni, Bashir Lone, Jasvinder Singh, Pankaj Kumar, Sumeet Gairola, Shashank Singh, Prasoon Gupta

**Affiliations:** ^1^Natural Products and Medicinal Chemistry Division, Indian Institute of Integrative Medicine, Jammu, India; ^2^Academy of Scientific & Innovative Research (AcSIR), Ghaziabad, India; ^3^Cancer Pharmacology Division, Indian Institute of Integrative Medicine, Jammu, India; ^4^Plant Science Division, Indian Institute of Integrative Medicine, Jammu, India

**Keywords:** *Roscoea purpurea*, Astavarga, Zingiberaceae, labdane diterpenes, cytotoxic, coronarin k, coronarin L

## Abstract

The main objective of cancer treatment with chemotherapy is to kill the cancerous cells without affecting the healthy normal cells. In the present study, bioactivity-guided purification of the *n*-chloroform soluble fraction from the methanol extract of *Roscoea purpurea* resulted in the identification of two new labdane diterpenes: coronarin K (**1**) and coronarin L (**2**), along with eight known compounds, coronarin A (**3**), bisdemethoxycurcumin (**4**), kaempferol 3-*O*-methyl ether (**5**), kaempferol (**6**), fenozan acid (**7**), 3-(3-methoxy,4-hydroxyphenyl)-2-propenoic acid ferulic acid (**8**), caffeic acid (**9**), and gallic acid (**10**). The structural identification of new compounds (**1** and **2**) were determined by detailed analysis of 1D (^1^H and ^13^C) and 2D NMR (COSY, HSQC, and HMBC) spectroscopic data. The relative configurations of 1 and 2 were determined with the help of NOESY correlations and comparison of optical rotations with known labdane diterpenes, with established stereochemistry, while structure of known compounds was established by direct comparison of their NMR data with those reported in the literature. This is the first report of isolation of this labdane diterpenes and phenolic classes of secondary metabolites in *R. purpurea*. In the preliminary screening, the methanol extract and its fractions were tested for the cytotoxic activity against a panel of four cancer cell lines (A549, HCT-116, Bxpc-3, and MCF-7); extract and its chloroform fraction were found to be active against the lung cancer cell line, A-549, with IC_50_ value <25 μg/ml. Owing to the notable cytotoxic activity of the chloroform fraction, the compounds (**1–5**) were screened for their cytotoxicity against all the cell lines by MTT assay. Coronarin K, **1** showed significant cytotoxic potential against lung cancer cell lines (A-549), with IC_50_ value of 13.49 μM, while other compounds did not show activity below 22 μM.

## Introduction

In our continuing research program to discover bioactive natural products from natural resources, especially from high-altitude Himalayan endangered medicinal plants, with profound biological activities, our attention was focused on the rhizomes of *Roscoea purpurea*, commonly known as kakoli, which belongs to the family of Zingiberaceae that is abundantly available in alpine grassland, grassy hillsides, and stony slopes of central to eastern Himalaya from Uttarakhand to North East states, up to an altitude of 3,300 m. It is an essential ingredient of an important Ayurvedic preparation, known as *Astavarga*, which is a group of eight plants claimed to be useful for healing weakness, body overweight, bone fractures, high fever, and diabetic situations, as well as for healing vata, pitta, and rakta doshas (Dhyani et al., 2010). The groups of Astavarga plants are considered as a very excellent *Rasayana*, with health-promoting and rejuvenating properties, and are recognized to support our immunity and have the capacity for cell renewal (Balkrishna et al., 2012). *Astavarga* plants are also known to restore human health and work as strong antioxidants in our body (Mathur, 2003; Pandey, 2005; Sharma and Balkrishna, 2005). Among the eight Astavarga plants, *R. purpurea* is one of the essential ingredients of several herbal formulations like tonic and Chyawanprash. Traditionally, it is used for the cure of diabetes, diarrhea, hypertension, fever, immunostimulant, inflammation, etc. In Nepal, its tubers are boiled for an edible purpose and are also used in traditional veterinary medicine (Singh and Rawat, 2011). In view of its significance in the Ayurvedic medicinal system, substantial pharmacological and phytochemical works have not been carried out. Previous phytochemical studies on *R. purpurea* have described the isolation of two principal groups of compounds: steroids and phenolic derivatives (Misra et al., 2015; Srivastava et al., 2015; Rawat et al., 2016). To date, only a few compounds have been identified and quantified through high-performance liquid chromatography (HPLC) analysis from tubers of this plant by Singh and co-workers, and they are presumed to be associated with its potent antioxidant activity (Gopal et al., 2014; Rawat et al., 2014). Alcoholic extracts of the plant have shown *in vitro* anti-cancer (Srivastava et al., 2015), antioxidant (Rawat et al., 2016), and immunomodulatory activities (Sahu et al., 2010). In our preliminary pharmacological study, cytotoxic activity was found for the methanol extract of *R. purpurea* against lung cancer cell line at IC_50_ 25.71 (μg/ml). On bioassay-guided fractionation, activity was localized in a chloroform-soluble fraction. Further work led to the isolation of two new compounds, along with eight known compounds for the first time from this plant species. In the present paper, we have described isolation and structure elucidation of two new compounds from the rhizomes of *R. purpurea* and the anti-cancer activities of plant extract, fractions, and isolated compounds.

## Materials and Methods

### General Experimental Procedures and Chemicals

All NMR spectral data were obtained on a Bruker 400 MHz spectrometer. Chemical shifts (δ) were referenced to the residual solvent peak (CD_3_OD: ^1^H δ 3.30, ^13^C δ 49.0 ppm; CDCl_3_: ^1^H 7.26, ^13^C 77.0 ppm; DMSO-d_6_: ^1^H 2.50, ^13^C 39.5 ppm), and the reference point was TMS (δ_H_ and δ_C_: 0 ppm). ^1^H-^13^C NMR correlations were established from gradient-enhanced inverse-detected HMBC and HSQC experiments, respectively. HPLC purification was done on a 1260 Infinity II Agilent system, equipped with a photo diode array (PDA) detector. HPLC solvents were purchased from Merck (Mumbai)—and water used for plant extractions and HPLC analysis was obtained from—A10 Milli-Q Advantage water system (Millipore, France). Column chromatography was done using silica gel (100–200 mesh). Thin layer chromatography (TLC) analyses were performed on Merck Kieselgel (Aufoilen) 60 F_254_ plates. Potato Dextrose Agar (PDA), 1% sodium hypochlorite, lactophenol cotton blue (LPCB), and 15% Glycerol were purchased from Difco, United States. Dulbecco's modified eagle medium (DMEM), Rosewell Park Memorial Institute Medium (RPMI-1640), Fetal bovine serum (FBS,10%), Rhodamine 123 (Rh 123), DAPI (4′,6-diamidino-2-phenylindole, dilactate), DMSO, gentamicin sulfate, trypsin, EDTA, PBS penicillin, streptomycin, paclitaxel, mitomycin, doxorubicin, gemcitabine, and all other chemicals were purchased from Sigma–Aldrich (St. Louis, MO, United States). Tris buffer, glacial acetic acid, and trichloroacetic acid (TCA) used in the study were purchased from Himedia (Mumbai), Fisher Scientific (Mumbai), and Merck Specialties Private Ltd., (Mumbai), respectively.

### Plant Material

Rhizomes of *R. purpurea* Sm. (Syn: *Roscoea procera* Wall.) were collected in September 2013 from Dhanaulti, Mussorie in district Tehri Garhwal of Uttarakhand, India (between 2,500 and 3,200 m asl). The crude herb was authenticated by Dr. Sumeet Gairola, and the voucher specimen (CDR No. 4027) was deposited in the Crude Drug Repository of CSIR-IIIM, Jammu.

### Extraction and Isolation

**Figure 1:**
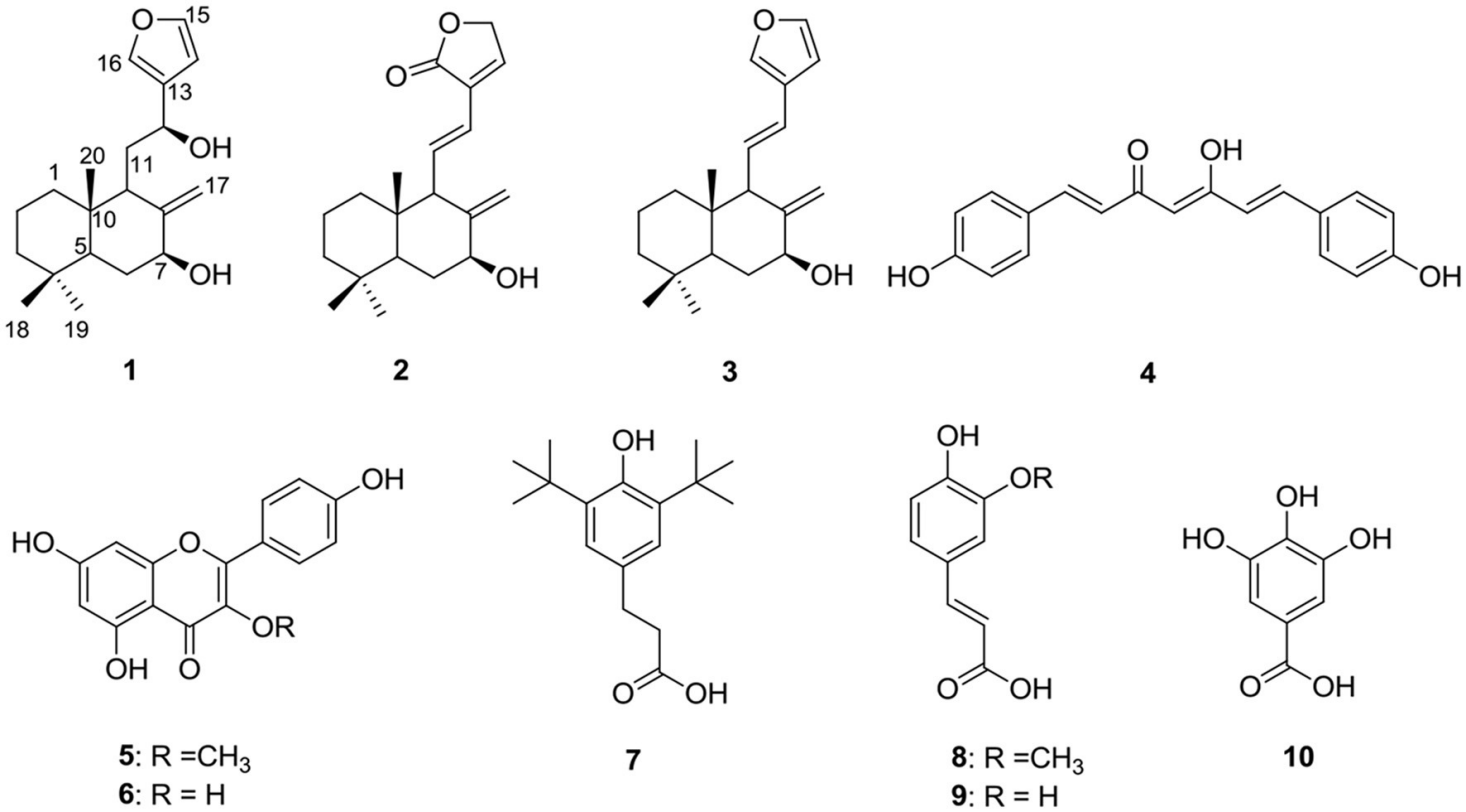
Structure of compounds from *R. purpurea* rhizomes (**1–10**).

Air-dried *R. purpurea* rhizomes (2.0 kg) were grounded into powder and extracted thrice (soaked for 24 h each), with 3 L of methanol. The extract was filtered—concentrated under reduced pressure (using rotavapor) at 40°C to yield a crude extract (305 g). Water (800 ml) was then added to dissolve the crude extract. The same amount of petroleum (pet) ether was used for solvent–solvent fractionation, and the upper layer (petroleum extract) was removed (40.1 g). The residue was further partitioned, using chloroform (165 g) and *n*-Butanol (62.5 g). The chloroform fraction (150 g) was purified by column chromatography (silica gel, 100–200 mesh), and eluted with a gradient of pet. ether-ethyl acetate (100:0 to 0:100, 500 ml collected volumes of each fraction), and concentrated, giving 20 combined fractions (Fr.1–Fr.20) based on the TLC profile. Fraction 2 (3.0 g) was further chromatographed over silica gel, with n-hexane-ethyl acetate (100:0 to 80:20, 100 ml collected volumes of each fraction), to afford four subfractions (Fr. 2A-2D). White crystals were formed in Fr. 2B-yielded 1.0 g of compound **3** (1.0 g), which was detected on TLC [Pet. Ether-EtOAc (97:3)] as tan-brown spot by charing the dried TLC in the anisaldehyde reagent. Further, purification of fraction F-4 (6.45 g) on silica gel (60–120 mesh, 200 g), using a gradient of n-hexane–EtOAc (95 to 55%), afforded 25 fractions (100 ml each), and these fractions were separated into six subfractions (F-4A to F-4F) on the basis of similarities in their TLC profiles. Purification of fraction F-4B (1.2 g) on silica gel (230–400 mesh, 40 g), using n-hexane–EtOAc (94:6), afforded compound **1** (0.6 g) as a white crystals. Also, purification of fraction F-6 (1.1 g), over silica gel (60–120 mesh, 170 g), using n-hexane–EtOAc (80:20), afforded compound **2** (70 mg) as white powder. Moreover, fraction F-10 (10.2 g) was loaded on a silica gel column (100–200 mesh, 300 g) and eluted, using chloroform as a mobile phase, afforded 6 subfractions (F-10A–F-10F). Subfraction F-10C (3.2 g) contains two major compounds, which, on further purification, give rise to compound **4** (0.4 g) and compound **5** (1.01 g), respectively. Compound **6** (45 mg) was solidified as a yellow powder in fraction F-10E. Fr. 6C (1 g) was further purified by column chromatography (silica gel), eluted with ethyl acetate (1–5%) in petroleum ether, giving 10 fractions of volume 50 ml each. White crystals were formed in the last five fractions, which were purified by washing with HPLC grade hexane-yielded compound **7** (0.32 g). Fraction F-15 (3.7 g) was chromatographed on silica gel (60–120 mesh, 120 g), eluted with a gradient of chloroform and methanol 95–5%, and yielded F-15A to F-15J. Fraction F-15E was stored in a refrigerator to yield **10** (62 mg). Column chromatography of F-15F (1.1 g), using EtOAc–MeOH (97:3), as eluent over silica gel (230–400 mesh, 30 g), afforded **8** (28 mg) and **9** (42 mg), respectively. The chemical structures of all the isolated compounds are given in Figure 1.

### Anti-cancer Activity

#### Cell Lines, Cell Culture, Growth Conditions, and Treatment

Different human cancer cell lines—the lung (A549), the colon (HCT-116), the breast (MCF-7), and the pancreas (Bxpc-3)—were obtained from the National Cancer Institute, Frederick (NCI), United States. Bxpc-3 and HCT-116 cell lines were maintained in DMEM, and A549, MCF-7 cell lines were maintained in RPMI. FBS (10%), penicillin (100 units/ml), streptomycin (100 μg/ml), and glutamine (2 mM) were used for supplementation. Cells were grown at 37°C in 5% CO_2_ (incubator, Thermo Scientific, United States), with 98% humidity. The concentration of DMSO added to the test materials was maintained at <0.2%.

#### Cell Proliferation Assay

Colorimetric MTT assay was used to carry out cell viability studies (Kumar et al., 2013). The plant extract and isolates were screened at different concentrations with a maximum concentration of 100 μM. The cells were rinsed and incubated with 20 μl of MTT dye after treatment at different time intervals. The medium was removed, and DMSO was added to each well after incubation for the solubility of purple formazan crystals. Microplate Reader (BioTek Synergy HT) was used to measure the absorbance at 570 nm. GraphPad Prism Software (La Jolla, CA, United States) was used to determine cell viability percentage and IC_50_ values.

#### Detection of Reactive Oxygen Species (ROS) Accumulation

Lung cancer cell line (A-549) seeded in six-well plates (2 × 10^5^) was treated with coronarin K **1** (7, 13, 18 μM) and incubated for 48 h. H_2_O_2_ was added to a well containing a medium for 2 h before termination. The cells were further incubated with 10 μM DCF for 30 min at 37°C in the dark. The cells were washed with PBS and analyzed with a fluorescence microscope (Olympus-1X53 magnification). The fluorescent intensity was measured at the emission and excitation wavelength of 480/530 nm (Banskota et al., 2015).

## Results and Discussion

### Identification of Compounds

The dried and powdered rhizomes of *R. purpurea* were extracted with methanol and dried at 40–45°C. The obtained crude extract was sequentially fractionated with pet ether, CHCl_3_, and *n*-butanol. The chloroform soluble fraction was purified, using chromatographic techniques, which afforded two new compounds (**1**–**2**), together with 8 known compounds. The structures of both novel secondary metabolites were established, using detailed NMR spectroscopic studies, whereas the structures of known compounds were identified in comparison with the previously reported literature data. The ^1^H and ^13^C NMR spectra of compounds **1** and **2** have some common structural features of diterpenes based on the NMR data and gave a positive copper acetate test for diterpenes.

**Figure 2:**
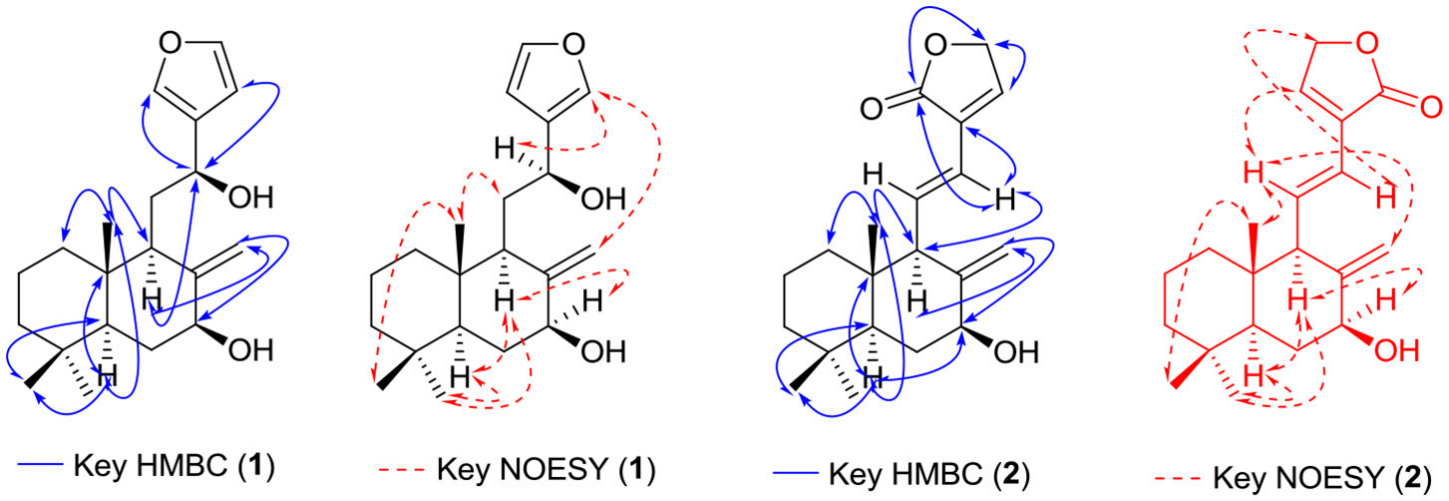
Key HMBC and NOESY correlations of compounds **1** and **2**.

Coronarin K (**1**) was isolated as a white crystal. The molecular formula of coronarin K (**1**), C_20_H_30_O_3_, was established from the ESIMS of the [M-H]^−^ ion at m/z 317.10, with six degrees of unsaturation. On analyzing the ^13^C, the NMR data showed the presence of three C=C bonds (δ_C_ 144.6, 130.1, 108.5, 143.4, 138.5, and 110.3), and three methyls (δ_C_ 33.6, 23.6, and 17.3), which revealed six double-bond equivalents and indicated the presence of one exomethylene and one furan ring in the molecule and suggested that **1** was labdane-type diterpene. A detailed analysis of the ^1^H NMR data (Table 1) showed the existence of a furan ring, presence of three olefinic proton signals at δ_H_ 6.43 (1H, s), 7.40 (1H, d, *J* = 1.2 Hz), and 7.39 (1H, d, *J* = 1.2 Hz), based on HSQC correlations to the olefinic carbon signals at δc 108.5 (C-14), 138.5 (C-15), and 143.4 (C-16), respectively. Furthermore, the presence of exocyclic methylene was revealed by the observation of ^1^H proton signals at δ_H_ 5.05 (1H, s) and 4.77 (1H, s), corresponding to δc 110.3 (C-17). The observed UV λmax at 238 nm was inconsistent with this assignment (Itokawa et al., 1988). HMBC correlations from the gem dimethyl signal at δ_H_ 1.02 (Me-18) and 1.22 (Me-19) to both C-4 (δ_C_ 34.5), a quaternary carbon at C-5 (δ_C_ 57.3), and to each other's carbon resonance-established methyl substitutions of C-4 (Figure 2).

**Table 1:** ^1^H NMR and ^13^C NMR data of compounds **1**^a^ and **2**^a^.

**Position**	**Compound 1**			**Compound 2**		
	**δ_**C**_**	**δ_**H**_ (mult, *J* = Hz)**	**COSY**	**δ_**C**_**	**δ_**H**_ (mult, *J* = Hz)**	**COSY**
1	41.9	1.76 (dt, 12.0, 2.9 Hz)	2	43.8	1.36, 1.03 (m)	2
		1.18 (dt, 12.0, 3.0 Hz)				
2	19.5	1.62, 1.51 (m)	1, 3	19.2	1.52, 1.36 (m)	1, 3
3	43.8	1.38, 1.20 (m)	2	44.0	1.18 (m)	2
4	34.5	–		34.5	–	
5	57.3	1.18 (d, 1.4 Hz)	6	56.8	1.12 (d, 1.3 Hz)	6
6	47.6	2.40 (d, 2.0 Hz)	5, 7	46.5	2.39 (d, 2.0 Hz)	5, 7
7	69.4	4.41 (bs)	6	68.9	4.33 (d, 2.9 Hz)	6
8	144.6	–		145.2	–	
9	52.6	2.20 (d, 10.2 Hz)	11	62.6	2.41 (d, 10.0 Hz)	11
10	40.5	–		40.3	–	
11	32.7	1.84 (dd, 10.0, 4.1 Hz)	9, 12	135.9	6.88 (dd, 15.8, 9.8 Hz)	9, 12
12	65.0	4.72 (dd, 4.0, 8.4 Hz)	11	121.0	6.06 (d, 15.6 Hz)	11
13	130.1	–		129.4	–	
14	108.5	6.43 (bs)	15	142.6	7.10 (s)	
15	138.5	7.39 (bs)	14	172.2	–	
16	143.4	7.40 (s)		69.6	4.87 (s)	
17	110.3	5.05, 4.77 (bs)		112.0	4.72, 4.74 (bs)	
18	33.6	1.02 (s)		33.6	1.03 (s)	
19	23.6	1.22 (s)		23.8	1.16 (s)	
20	17.3	1.18 (s)		18.0	1.12 (s)	

a *Recorded in CDCl_3_ at 400 MHz (TMS as internal standard), chemical shifts, multiplicity, and coupling constants (J, Hz) were assigned by means of ^1^H, ^13^C NMR, and 2D NMR data*.

The backbone of the bicyclic ring system was established, using detailed analysis of HMBC correlations from the three methyl signals: δ_H_ 1.02, 1.22, and 1.18, which obtained as a singlet in the ^1^H NMR spectrum and showed HMBC correlation with the H_3_-18 and H_3_-19 to C-3, 4, 5; H_3_-20 to C-1, 5, 8, and 10. The ^1^H NMR signals of the exocyclic methylene (δ_H_ 5.05, 4.77, δC 110.3) revealed strong HMBC correlations with H_2_-17 to C-7, 8, 9. The methylene H-9 also showed HMBC to C-5, 7, 10, 17, 20. Along with these, a correlation from H-9 to the oxymethine carbon (δ_C_ 65.0) is also revealed by HMBC. The presence of a decalin ring system was established with COSY correlations indicating a coupling from H_2_-6 to H-5 and H-7 and from H_2_-1 to H_2_-2 and H_2_-3.

**Figure 3:**
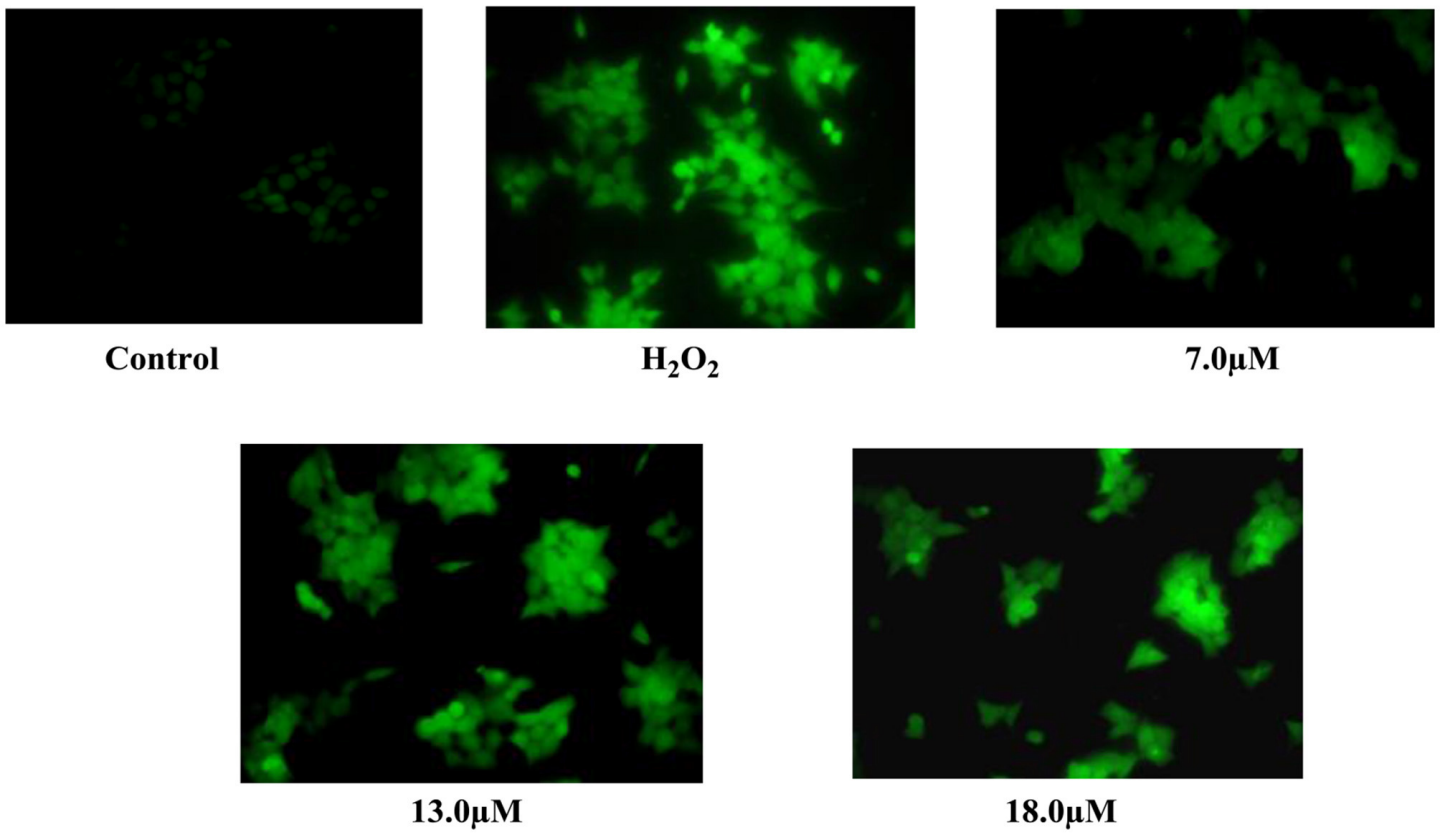
Effect of coronarin K (1) on cellular and nuclear morphology of A-549 in ROS production assessed from the oxidation of DCFDA by H_2_O_2_ through fluorescence microscopy at 48-h posttreatment.

All above HMBC and COSY correlations established connection of the bicyclic ring with furan ring. The other signal to be assigned was a methylene group CH_2_-11 (δ_H_ 1.84; δ_C_ 32.7). HMBC correlations from the H_2_-11 signal to C-12, 13 and H-12 to C-14 and 16 confirmed the connection of the C-9 to C-10 and C-12 to C-13 furan ring. Thus, the structure of **1** was characterized as Coronarin K, a new labdane diterpene. The relative signs of **1** were established by NOE correlations observed in the NOESY experiment (Figure 3) and observed optical rotation +23.3° (c.23, CHCl_3_), which is consistent with published data for a similar skeleton (Itokawa et al., 1988). NOE correlations from H_3_-20 to H-11, and H_3_-18, together with correlations from H_3_-19 to H-5, H-9; H-5 to H-9; H-9 to H-8; H_2_-17 and H-12 to H-16, established the decalin ring system. Thus, the structure of coronarin K (**1**) is defined as 5S^*^, 7S^*^, 9R^*^, 10S^*^, 12S.

Coronarin L (**2**) was isolated as a white solid. The molecular formula of Coronarin L (**2**) was established as C_20_H_28_O_3_, based on the ESIMS and NMR data. The cyclic lactone group in **2** was confirmed in the IR spectrum (1,750 cm^−1^) by the presence of C=O-stretching bands **(**Itokawa et al., 1988). NMR data comparison (Table 1) revealed that **2** was structurally similar to **1**, except for the presence of a diene system [δ_H_ 6.88 (1H, dd, *J* = 15.8, 9.8 Hz Hz), δ_C_ 135.9 (C-11); δ_H_ 6.08 (1H, d, *J* = 15.6 Hz), δ_C_ 121.0 (C-12)] in place of oxymethine at C-12 as it was missing in ^13^C spectrum. Furthermore, changes in the proton and carbon chemical shifts of the furan ring suggested oxidation of the C-14–C-15 double bond to a carbonyl group. HMBC correlations from the methine signals at δ_H_ 4.87 (2H, s, H-16) to C-15 (C=O) and C-14 (δ_H_ 7.10, s), together with HMBC correlation with diene C-12, confirmed the presence of a lactone ring at C-12 adjacent to a double bond. A COSY correlation, together with the large ^1^H coupling constant (*J* = 15.8 Hz), between H-11 and H-12, established the E geometry of the C-11-C12 double bond. NOE correlations in **2** (Figure 2) and observed optical rotation +36.9° (c.20, CHCl_3_) were identical to that of 1; therefore, the structure of coronarin L (**2**) was defined as 5S^*^, 7S^*^, 9R^*^, 10S^*^.

Compound **3** was another labdane diterpene isolated as white crystals. The molecular formula was confirmed as C_20_H_28_O_2_ based on the significant signal observed in the HR-ESIMS spectrum at m/z 301.2167 [M+H]^+^. Compound **3** carries a similar chemical skeleton as compound **1** based on comparison of NMR data. After complete ^1^H and ^13^C and 2D NMR analysis, the structure was confirmed as Coronarin A, previously reported from *Hedychium coronarium* (Itokawa et al., 1988). The structures of the known compounds **4-10** (Figure 1) were identified as bisdemethoxycurcumin (**4**) (Dairam et al., 2007), kaempferol 3-*O*-methyl ether (**5**) (Hammami et al., 2004), kaempferol (**6**) (Gupta et al., 2007), fenozan acid (**7**) (Jaivel et al., 2014), 3-(3-methoxy,4-hydroxyphenyl)-2-propenoic acid (ferulic acid), (**8**) (Diaz et al., 2012), caffeic acid (**9**) (Yoshioka et al., 2004), and gallic acid (**10**) (Zucca et al., 2013) by direct comparison of ESIMS, ^1^H and ^13^C NMR data with the previously reported literature data. All the known compounds **4-10** were isolated for the first time from this species.

### Anti-cancer Activity

Secondary metabolites, which were isolated from the plants of the Zingiberaceae family, have wide use as a new pharmacophore in anti-cancer drug discovery. In our bio-assay-guided isolation of novel anti-cancer agents from *R. purpurea*, the methanol extract and respective fractions were screened for their anti-cancer activity, using the MTT assay. The methanol extract and its CHCl_3_ fraction showed cytotoxic activity against lung cancer cell line (A549) at IC_50_ 27.71 and 21.35 (μg/ml), respectively. After purification of chloroform fraction, compounds 1–10 were isolated. Only compounds **1–5** were evaluated for different cancer cell lines. Among the tested compounds, Coronarin K (**1**) showed prominent activity against lung cancer cell line (A-549) and also showed moderate cytotoxic activity against colon cancer cell line HCT-116 (IC_50_ value of 26.03 μM) but had no effect on Bxpc-3 and MCF-7 in acceptable limits. Compounds **2** and **3** were ineffective against all the cell lines except pancreatic cancer cell line (Bxpc-3) at IC_50_ value of 26.03 μM. The remaining tested compounds displayed less activity (Table 2). In a further study, the A-549 cells were treated with compound **1** at 7.0, 13.0, 18.0 μM, and ROS generation was detected in 48 h. Hydrogen peroxide was taken as a positive control. After exposing the cells with compound **1**, the fluorescence intensity of DCF was increased in a dose-dependent manner. The results showed that compound **1** exhibited prominent cytotoxicity against human lung cancer cell line (A-549), with IC_50_ value of 13.49 μM (Figure 3) and thereby confirmed the ROS generation is crucial to cell death.

**Table 2:** Cytotoxicity of extract, fraction, and isolated compounds (1–5)^a^.

**Compound**	**IC**_**50**_, **μM, in different human cancer cell lines**			
	**A-549**	**HCT-116**	**Bxpc-3**	**MCF-7**
MeOH extract (μg/ml)	25.71 ± 0.21	90.92 ± 0.46	62.72 ± 1.23	48.96 ± 2.36
CHCl_3_ fraction (μg/ml)	21.35 ± 0.83	>100	68.95 ± 3.21	46.64 ± 0.42
1	13.49 ± 0.62	26.03 ± 1.46	56.70 ± 2.17	56.24 ± 0.83
2	33.78 ± 1.37	>50	56.83 ± 1.92	49.84 ± 2.61
3	61.80 ± 2.82	>50	22.83 ± 1.47	>50
4	>50	>50	68.15 ± 2.41	>50
5	>50	>50	>50	>50
Paclitaxel	6.2 ± 0.20	8.6 ± 0.04	5.46 ± 0.74	3.81 ± 0.32

a *Data are mean ± SD*.

## Conclusion

In the present study, by the cytotoxic activity-guided isolation of *R. purpurea* extract, we have made an unusual finding of two new labdane diterpenes coronarin K (**1**) and coronarin L (**2**), together with the other known compounds **3–10**. All the compounds have been isolated physically for the first time from this plant. Furthermore, this is the first report of the natural origin (Fenozan acid **7**) of a synthetic *di*-*tert*-butyl derivative in plants. In addition to this, we have evaluated the anti-cancer activity of extract, chloroform fraction, and isolated compounds. Among all the tested compounds, only coronarin K (**1**) showed prominent anti-cancer activity against lung cancer cell line (A-549). Our study also suggest that the antiproliferative effect of the compounds is due to ROS generation and also led to the impaired cell migration. Finally, our study strongly suggests that screening and detailed chemical study of endangered *Astavarga* plants would be valuable.

## Data Availability Statement

The original contributions presented in the study are included in the article/Supplementary Material, further inquiries can be directed to the corresponding author/s.

## Author Contributions

PG conceptualized and wrote the paper. VS and BL performed isolation and characterization of compounds. PK and SG helped in the collection and the identification of the plant material. JS and SS performed the anti-cancer activity. All the authors read and approved the final manuscript.

## Conflict of Interest

The authors declare that the research was conducted in the absence of any commercial or financial relationships that could be construed as a potential conflict of interest.

## Supplementary Material

Spectroscopic data of compounds **1** and **2**, including 1D (^1^H and ^13^C), 2D (^1^H-^1^H COSY, HSQC, HMBC, and NOESY), NMR and ESIMS.
