# Corrigendum: Coronarin K and L: Two Novel Labdane Diterpenes From *Roscoea purpurea*: An Ayurvedic Crude Drug

**DOI:** 10.3389/fchem.2021.798461

**Published:** 2021-11-23

**Authors:** Venugopal Singamaneni, Bashir Lone, Jasvinder Singh, Pankaj Kumar, Sumeet Gairola, Shashank Singh, Prasoon Gupta

**Affiliations:** ^1^ Natural Products and Medicinal Chemistry Division, Indian Institute of Integrative Medicine, Jammu, India; ^2^ Academy of Scientific & Innovative Research (AcSIR), Ghaziabad, India; ^3^ Cancer Pharmacology Division, Indian Institute of Integrative Medicine, Jammu, India; ^4^ Plant Science Division, Indian Institute of Integrative Medicine, Jammu, India

**Keywords:** *Roscoea purpurea*, Astavarga, *Zingiberaceae*, labdane diterpenes, cytotoxic, coronarin K, coronarin L

In the original article, there was an error in affiliation 1. Instead of “Natural Product and Medicinal Chemistry Division, Indian Institute of Integrative Medicine, Jammu, India” it should be “Natural Products and Medicinal Chemistry Division, Indian Institute of Integrative Medicine, Jammu, India”.

In the original article, there was an error in affiliation 2. Instead of “Academy of Scientific & Innovative Research (AcSIR), Council of Scientific and Industrial Research, New Delhi, India,” it should be “Academy of Scientific & Innovative Research (AcSIR), Ghaziabad, India.”

The authors apologize for this error and state that this does not change the scientific conclusions of the article in any way. The original article has been updated.

