# Supplementary material for: Coronarin K and L: Two Novel Labdane Diterpenes From Roscoea purpurea: An Ayurvedic Crude Drug
Source: Front Chem. 2021 Apr 21;9:642073. doi: 10.3389/fchem.2021.642073 (PMC8097143; doi:10.3389/fchem.2021.642073)
Supplement: Supplementary file 1 [file Data_Sheet_1.docx]

**Supporting Information**

**Coronarin K and L: Two Novel Labdane Diterpenes from Roscoea purpurea: An Ayurvedic Crude Drug**

Venugopal Singamaneni^1^, Bashir Lone^1^, Jasvinder Singh^2,3^, Pankaj Kumar^4^, Sumeet Gairola^2,4^, Shashank Singh^2,3^, Prasoon Gupta^1,2,^*

*^1^Natural Product Chemistry Division, Indian Institute of Integrative Medicine, Canal Road, Jammu-180001, India*

*^2^Academy of Scientific & Innovative Research (*AcSIR*), CSIR, New Delhi-110025, India*

*^3^Cancer Pharmacology Division, Indian Institute of Integrative Medicine, Canal Road, Jammu-180001, India*

*^4^Plant Science Division, Indian Institute of Integrative Medicine, Canal Road, Jammu-180001, India*

------------

* To whom corresponding should be addressed.

Dr. Prasoon Gupta Ph.D.

Principal Scientist

Indian Institute of Integrative Medicine

Canal Road, Jammu-180001

Office: +91(191) - 2585006-13 Ext-485

Fax: 0191-2569333

Mail: [guptap@iiim.ac.in](mailto:guptap@iiim.ac.in)

**Supporting Information**

**Figure S1.** ^1^H NMR (400 MHz, CDCl_3_) spectrum of compound **1**

**Figure S2.** ^13^C NMR (100 MHz, CDCl_3_) spectrum of compound **1**

**Figure S3.** ^13^C NMR (DEPT) (100 MHz, CDCl_3_) spectrum of compound **1**

**Figure S4.** HMBC spectrum of compound **1**

**Figure S5.** HSQC spectrum of compound **1**

**Figure S6.** COSY spectrum of compound **1**

**Figure S7**. ESI-MS Spectrum of compound **1**

**Figure S8.** ^1^H NMR (400 MHz, CDCl_3_) spectrum of compound **2**

**Figure S9.** ^13^C NMR (100 MHz, CDCl_3_) spectrum of compound **2**

**Figure S10.** ^13^C NMR (DEPT) (100 MHz, CDCl_3_) spectrum of compound **2**

**Figure S11.** HMBC spectrum of compound **2**

**Figure S12.** HSQC spectrum of compound **2**

**Figure S13.** COSY spectrum of compound **2**

**Figure S14**. ESI-MS Spectrum of compound **2**

**Figure S1.** ^1^H NMR (400 MHz, CDCl_3_) spectrum of compound **1**

**Figure S2.** ^13^C NMR (100 MHz, CDCl_3_) spectrum of compound **1**

**Figure S3.** ^13^C NMR (DEPT) (100 MHz, CDCl_3_) spectrum of compound **1**

**Figure S4.** HMBC spectrum of compound **1**

**Figure S5.** HSQC spectrum of compound **1**

**Figure S6.** COSY spectrum of compound **1**


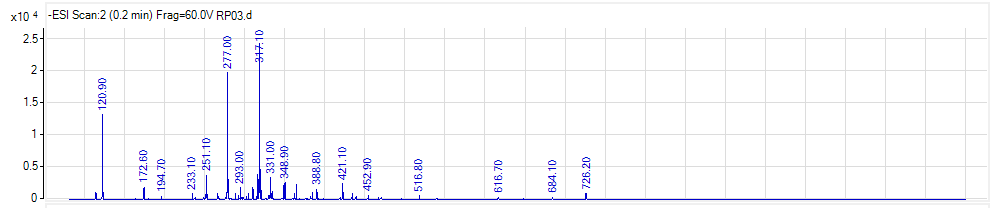


**Figure S7**. ESI-MS Spectrum of compound **1**


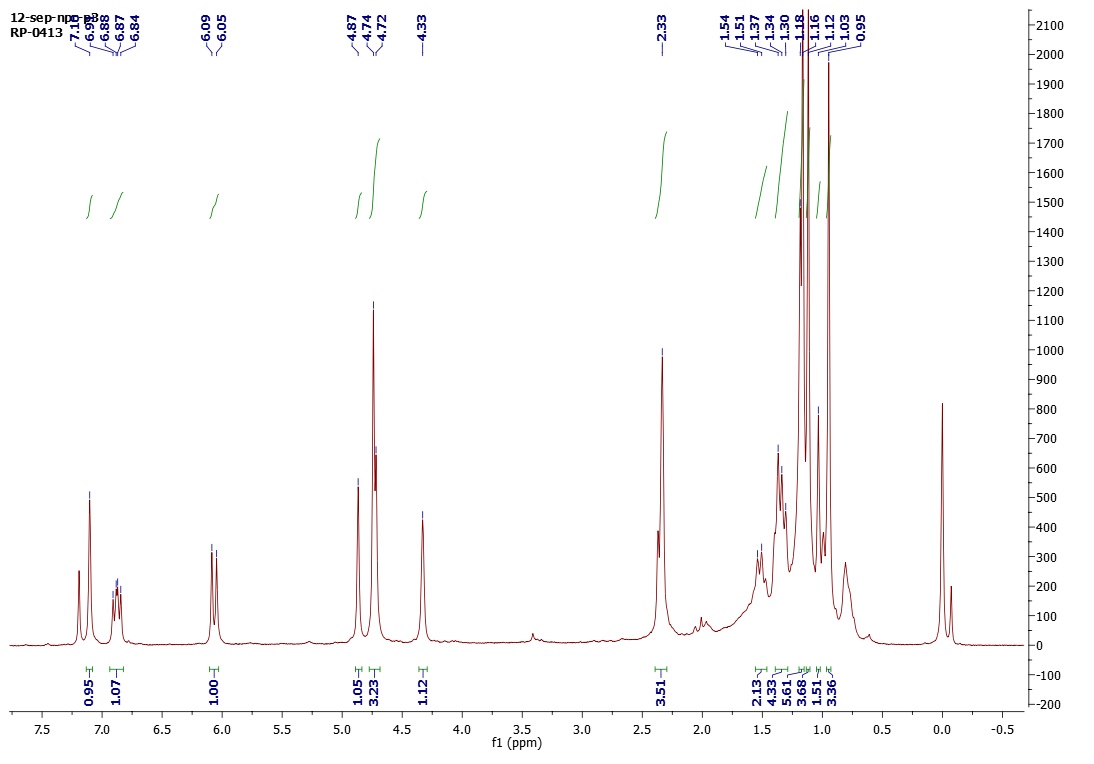


**Figure S8.** ^1^H NMR (400 MHz, CDCl_3_) spectrum of compound **2**

**Figure S9.** ^13^C NMR (100 MHz, CDCl_3_) spectrum of compound **2**

**Figure S10.** ^13^C NMR (DEPT) (100 MHz, CDCl_3_) spectrum of compound **2**

**Figure S11.** HMBC spectrum of compound **2**

**Figure S12.** HSQC spectrum of compound **2**

**Figure S13.** COSY spectrum of compound **2**

^
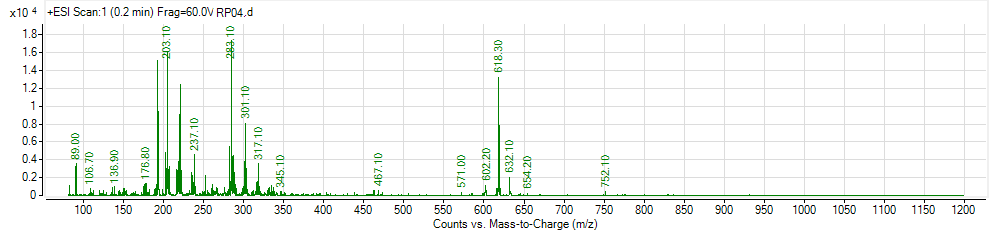
^

**Figure S14**. ESI-MS Spectrum of compound **2**
